# Botanical Description of British Plants

**Published:** 1807-09

**Authors:** 


### *Botanical Description of British Plants.*

[ Continued from Vol. xviii. page 169—174. ]

7. COCHLEARIA. *C. officinalis.—C. hortensis. C. rotundifolia.*

*Ang.* Scurvy grass. Scrooby grass.

*Gen. Desc.* Pouch notched, turgid, rough, many seeded; valves bulging, blunt.

*Spec. Desc. Root leaves* heart-circular, entire, veined, on long leaf-st. fleshy. *Stem-l.* oblong, sitting, a little indented. *Stem* angular. *Pet.* fleshy, clear white claws, greenish. *Pouch* v. slightly notched, smooth. *Partition* double. *Seeds* rough.—*Sea shore.* BL. April, May.

*Use.* It has an unpleasant smell, and a warm acrid bitter taste. Its principal virtue resides in an essential oil, separable in very small quantity by distillation with water, and so ponderous as to sink in the aqueous fluid; but of great volatility, subtility, and penetration: one drop dissolved in spirit, or received on sugar, communicates to a quart of wine, or other liquor, the smell and taste of scurvy grass.—*Lewis.* Scurvy grass is *antiseptic, (See Pringle's Exp.) attenuant, aperient,* and *diuretic,* and is said to open the obstructions of the viscera and remoter glands, without heating or irritating the system: it has long been considered the most effectual of all the *antiscorbutic* plants, for which we have the testimony not only of physicians, but of the most celebrated navigators who have experienced its beneficial effects at sea. *(Anson, Linschoten, &c. &c.)*

*&c.)* And it is worthy of remark, that it grows most plentifully in those high latitudes in either hemisphere, where the scurvy is most obnoxious. Its sensible qualities are sufficiently powerful to confirm this opinion. In *rheumatismus vagus,* called by Sydenham scorbutic rheumatism, consisting of wandering pains of long continuance, accompanied by fever, this plant, combined with arum and wood sorrel, is highly recommended both by Sydenham and Lewis. A remarkably volatile and pungent spirit, prepared from this herb, and known by the name of *Sp. antiscorbuticus, s. mixtura simplex antiscorbutica Drawizii,* was found by Werlof to be a useful remedy in *paralysis,* and other diseases requiring an active and powerful stimulant, given in the dose of thirty drops several times a-day. *(See Cullen M. M. v. ii.* 165.*)* But as an antiscorbutic, neither this nor the conserve promises so much benefit as the fresh plant eaten in the way of sallad, or the expressed juice, as directed in the Pharmacopœias. — *Woodville.* There are instances of whole ships' crews having been cured of the sea-scurvy by the use of this plant: and as it abounds with acid salts, there is no doubt that it resists putrefaction. The best mode of taking it is raw, in sallad. It is also *diuretic,* and useful in dropsies. The Highlanders esteem it a good stomachic.—*Lightfoot.* Its effects as an anti-scorbutic are universally known; and it is a powerful remedy in the *pituitous asthma,* and in what Sydenham calls the scorbutic rheumatism. It possesses a considerable degree of acrimony, which seems to reside in a very subtile essential oil: a distilled water and a conserve are prepared from the leaves, and its juice is prescribed along with that of oranges, by the name of antiscorbutic juices. It may be eaten in sallads. Notwithstanding that it is a native of the Sea-coast, it is cultivated in gardens without any sensible alteration of its properties. Cows eat it; horses, sheep, and goats refuse it.—*Withering.* In Iceland it is said that sheep eat it greedily, and fatten very greatly upon it, but their flesh acquires from it a nauseous flavor. *See Bergius M. M.* 557.

8. COCHLEARIA. *C. coronopus.*

*Ang.* Swine's cress. Scurvy grass.

*Gen. Desc. As above.*

*Spec. Desc. Leaves* wing-cleft. *Stem* depressed. *Root-leaves* prostrate; *leafits* cut along the fore edge, entire on the back edge. *Pouch* kidney-heart-shaped, furrowed and ridged. *Bunches* axillary. *Bloss.* white. *Cornfields, rubbish.* B. June, Aug.

*Use.*

*Use.* This plant was rendered famous some years ago, by its ashes being an ingredient in Mrs. Johanna Stephens's celebrated medicine for the stone and gravel: but unfortunately it has not supported its credit.—*Lightfoot.*

9. COCHLEARIA. C. *armoracia.*—*C. didyma. Raphanus rusticanus.*

*Ang.* Horse-radish.

*Gen. Desc.* As above.

*Spec. Desc. Root-leaves* spear-shaped, scolloped; *stem-leaves* snipt. *Flowers* white. *Var. Root-l. wing-cleft.*—Sides of ditches and rivers. BL. May.

*Use.* The root has long been received into the Mat. Med. and affects both the taste and smell with a quick penetrating pungency, but in certain vessels it contains a sweet juice, which sometimes exudes in little drops upon the surface: its pungent matter is of a very volatile kind, being wholly dissipated by drying.—*Lewis.* The root affords one of the most acrid substances of this order, and therefore proves a powerful stimulant, whether externally or internally employed. Externally, it readily inflames the skin and proves a *rubefacient*, that may be employed with advantage in *palsy* and *rheumatism*; and its application, if long continued, produces blisters. Internally it may be used with advantage for the cure of *hoarseness* which proceeds from an interrupted secretion of mucus: one drachm of the root, fresh scraped down, is enough for four ounces of water, to be infused in a close vessel for two hours, and made into a syrup with double its weight of sugar; of this syrup a tea-spoonful or two, swallowed leisurely, or at least repeated two or three times, has been found very suddenly effectual in relieving hoarseness.—This syrup is a good substitute for the juice of the *erisimum*, when the latter is not at hand, (*Cullen M. M. ii.* 167.) Received into the stomach, it stimulates, and promotes digestion; and therefore is a proper condiment for animal food. A portion of its infusion taken with a large draught of warm water readily proves *emetic*, and may be either employed as such by itself, or to assist the operation of other emetics. Infused in water and taken into the stomach, it proves *stimulant* to the nervous system, and is thereby useful in *palsy*: employed in large quantity it is heating to the whole body; and hereby is often useful in *chronic rheumatism*, whether arising from scurvy or other causes. Cut down, without bruising, into very small pieces, and swallowed without chewing, in a large quantity, to the amount of a table-spoonful every morning for a month together, together, it is said to have been found extremely useful in *arthritic* cases, *(Berg. M. M.* 559*)*; but these were probably of the rheumatic kind: employed in this manner, it seems, like the unbruised mustard seed, to give out in the stomach its subtle volatile parts, that stimulate without inflaming. It is also a powerful *diuretic,* and therefore useful in *dropsy:* in this manner, by promoting urine and perspiration, it has long been known as one of the most powerful antiscorbutics. *Cullen M. M. v. ii. p.* 169.—The root scraped is in common use at our tables as a condiment for fish, roast beef, &c. and it is used for many other culinary purposes. In *paralytic* and *dropsical* cases it is an useful stimulant and diuretic: a strong infusion of it excites vomiting.—An infusion of it in cold milk, makes one of the safest and best *cosmetics.*—Horses, cows, sheep, goats, and swine refuse it.—*Withering.*

Tetradynamia. Siliquosa.

10. Cardamine. *C. pratensis.—Nasturtium pratense.*

*Ang.* Common lady's smock. Cuckow-flower.

*Gen. Desc.* Pod long, r. edged, opening with a jerk. Valves rolling back, parallel to the membranaceous partition. Summit a knob, entire. Calyx rather open.

*Spec. Desc. Leafits* of root leaves roundish; of *Stem leaves,* lower egg-shaped; higher spear-shaped; upper strap-shaped; entire. *Flowers* large, purplish red.—*Meadows.* Bl. May.

*Use.* The flowers have a place in the Materia Medica of the British Pharmacopœias upon the authority of Sir George Baker, who recommends them as an *antispasmodic* remedy. *See Med. Trans. ii.* 442. He relates five cases, where these flowers were successfully employed; and in a P. S. to the 2d edit. he says, "Since the first edition, I have seen several instances of flores cardamines in convulsive disorders." In epilepsy, however, this remedy has generally been found unsuccessful. The dose of the powdered flowers is from half a drachm to two drachms.—*Woodville.* The virtue of these flowers in hysteric and epileptic cases was first mentioned by Ray; and since then by Dr. Baker, *(Med. Tr.)* The dose is from twenty to ninety grains twice a day. In the family of the Rev. Mr. Gregor, in Cornwall, the flowering tops of this plant have been used for some generations in the cure of epileptic fits with success; whereas our medical people use only the flowers. Can this account for the different success? Sheep and goats eat it; cows are not fond of it; horses and swine refuse it. *Withering.* 11.

11. Cardamine. *C. amara.*

*Ang.* Bitter cresses, or lady's smock.

*Gen. Desc.* As above.

*Spec. Desc.* *Leaves* winged; *suckers* from the bosom of the leaves, round, crooked. *Leafits* angular, sitting, thinly serrated. *Stem* angular, strong, almost woody. *Bunches* terminating, lateral. *Bloss.* white; *anthers* purple. *Near water.* Bl. April, May.

*Use.* The leaves are pungent, bitter, and aromatic, in so great a degree as to promise very considerable medical uses.—*Withering.* The young leaves, though acrid and bitterish, do not taste amiss in sallads; *Lightfoot.* And in Lancashire they are much used for that purpose.—*Caley.* Sheep eat it; cows are not fond of it.—*Linn.*

12. Sisymbrium. *S. nasturtium.* *Nasturtium aquaticum.*

*Ang.* Water cresses.

*Gen. Desc.* Pod cylindrical, opening; valves straightish, about the length of the partition. Calyx and blossom expanding.

*Spec. Desc.* *Pods* declining, short. *Leaves* winged; *leafits* egg-shaped. *Bloss.* white, terminating. *Springs, brooks, rivulets.* Bl. June, July.

*Use.* The leaves have a moderately pungent taste, and a quick penetrating smell. Their antiscorbutic qualities have been long generally acknowledged by Physicians. They are also supposed to purify the blood and humours, and to open visceral obstructions; they are nearly allied to scurvy grass, but more mild and pleasant; and therefore are frequently eaten as sallad. The juice of this plant is directed, in the Pharm. with that of scurvy grass and seville oranges.—*Woodville.*

This plant is an excellent antiscorbutic and stomachic, with less acrimony than the scurvy grass: it is an ingredient in the antiscorbutic juices.—It is very universally used as an early and wholesome spring sallad.—*Withering.* It is said to be useful in jaundice and other visceral obstructions—*Lightfoot.*

( To be continued. )

